# The Impact of a Family History of Alcoholism on the Relationship Between Age at Onset of Alcohol Use and DSM–IV Alcohol Dependence: Results From the National Longitudinal Alcohol Epidemiologic Survey

**Published:** 1998

**Authors:** Bridget F. Grant

**Affiliations:** Bridget F. Grant, Ph.D., Ph.D., is the chief of the Biometry Branch, Division of Biometry and Epidemiology, National Institute on Alcohol Abuse and Alcoholism, Bethesda, Maryland

**Keywords:** family AODU (alcohol and other drug use) history, AOD dependence, AOD use initiation, prevalence, age, disease onset, risk assessment, risk factors, diagnostic criteria, survey, longitudinal study

## Abstract

Both the age at onset of alcohol use and a family history of alcoholism can influence a person’s risk of becoming alcohol dependent. The relationship between lifetime alcohol dependence, age at first alcohol use, and a family history of alcoholism was investigated using data obtained in the 1992 National Longitudinal Alcohol Epidemiologic Survey. This analysis demonstrated that regardless of the family history of alcoholism, respondents with an earlier age of drinking onset were more likely to become alcohol dependent compared with respondents with a later age of drinking onset. Among all age, race, and gender subgroups studied, however, people with a family history of alcoholism had a higher prevalence of lifetime alcohol dependence than did people without such a history.

Various factors can influence a person’s risk of developing alcohol dependence during his or her lifetime, including the age at which alcohol use first occurred and a family history of alcoholism. Epidemiologic analyses have found that people who started drinking at age 14 and younger are approximately four times as likely to become alcohol dependent as are those who began drinking at age 20 and older (Grant and Dawson 1997). Similarly, numerous studies have demonstrated that first-degree relatives of alcoholics are two to seven times more likely than people with nonalcoholic relatives to develop problems with alcohol at some time in their lives (National Institute on Alcohol Abuse and Alcoholism [NIAAA] 1997). This Epidemiologic Bulletin explores the relationship between lifetime alcohol dependence and the age at onset of alcohol use as a function of a family history of alcoholism. The article includes an analysis of the U.S. drinking population, classified by race and gender (i.e., race-gender subgroups). This study extends the findings of an earlier study that investigated the relationship between age at onset of drinking and alcohol use disorders (Grant and Dawson 1997).

## Background and Procedures

Lifetime prevalence estimates of alcohol dependence in this study were based on the 1992 National Longitudinal Alcohol Epidemiologic Survey (NLAES), a nationwide household survey sponsored by NIAAA (Grant et al. 1994). The survey consisted of face-to-face interviews with 42,862 respondents, age 18 and older, in the contiguous United States and the District of Columbia. The household response rate for the NLAES was 91.9 percent, and the person response rate was 97.4 percent. Fieldwork for the study was conducted by the Bureau of the Census.

The NLAES featured a complex multistage design (Massey et al. 1989). Primary sampling units (PSUs)^1^ were stratified according to sociodemographic criteria and were selected with probability proportional to size. The NLAES sample included approximately 200 PSUs, 52 of which were self-representing—that is, selected with certainty.^2^ Within PSUs, geographically defined secondary sampling units, called segments, were selected systematically for each sample. Because blacks experience higher rates of alcohol-related disease (e.g., liver cirrhosis) than do other population subgroups, oversampling of the black population was accomplished at this stage of sample selection.

Segments then were divided into clusters of approximately four to eight housing units, and all occupied housing units were included in the survey. Within each household, one person age 18 or older was randomly selected to participate in the survey. Oversampling of young adults (i.e., people ages 18 to 29) was accomplished at that stage of sample selection to increase the representation of this heavy-drinking population subgroup. Thus, young adults were sampled at a ratio of 2.25 percent to 1.00 percent.

Because of the complex survey design of the NLAES, variance estimation procedures that assume a simple random sample cannot be employed. Research has shown that the stratification and clustering strategies of the NLAES sample selection may result in standard errors much larger than those that would be obtained with a simple random sample of equal size. To take into account the NLAES sample design, all standard errors of the prevalence estimates presented here were calculated using SUDAAN (Research Triangle Institute 1997), a software program that uses appropriate statistical techniques to adjust for sample design characteristics.

### Measures

The definition of lifetime alcohol dependence was based on the diagnostic criteria of the *Diagnostic and Statistical Manual, Fourth Edition* (DSM–IV) (American Psychiatric Association 1994). DSM–IV-based diagnoses were established using the Alcohol Use Disorders and Associated Disabilities Interview Schedule (AUDADIS), a fully structured psychiatric interview designed to be administered by trained interviewers who are not clinicians (Grant and Hasin 1992). The AUDADIS includes an extensive list of symptom questions that operationalize the DSM–IV criteria for alcohol dependence.

The AUDADIS diagnosis of alcohol dependence satisfied the clustering and duration criteria of the DSM–IV definition. The criteria of the DSM–IV include the requirement for a clustering of symptoms within any 1-year period. The duration criterion is defined as the repetitiveness with which symptoms must occur to be counted as positive toward a diagnosis. The duration criterion is represented by the terms “recurrent” and “persistent” that appear in the description of most of the dependence diagnostic criteria. Not only was the clustering criterion represented in past-year AUDADIS diagnoses of dependence, but also the corresponding past diagnoses (i.e., before the past year) were measured as syndromes, or the clustering of the required number of symptoms necessary to achieve a diagnosis as follows: (1) at the same time, (2) continuously for at least 1 month, or (3) repeatedly for several months.

For the purposes of the current study, respondents were classified as having a lifetime alcohol dependence diagnosis if they had experienced an episode of dependence in the past year or at any time before the past year. Respondents classified with lifetime dependence included those with and without abuse diagnoses. To determine the reliability of alcohol dependence diagnoses established using this approach, an independent test-retest study was conducted in the general population before fielding the full NLAES (Grant et al. 1995). The analysis determined good reliabilities, with kappas of 0.76 and 0.73 for past-year and prior-to-past-year dependence diagnoses, respectively.

The age of drinking onset was ascertained by asking respondents how old they were when they first started drinking, not counting small tastes or sips of alcohol. Drinking onset data were collected from respondents who were classified as current drinkers (i.e., who had consumed at least 12 drinks in the past 12 months) and former drinkers (i.e., who had consumed at least 12 drinks in any 1 year of their lives but not during the year preceding the interview). The test-retest reliability of the drinking onset variable was good, with a kappa of 0.72 (Grant et al. 1995).

Measures selected as control variables included race (i.e., black versus white), gender, and family history of alcoholism (i.e., family-history positive [FHP] versus family-history negative [FHN]). The latter was ascertained through a series of questions that asked about different types of first-degree biological relatives (i.e., parents, children, and siblings). For each type of relative, the respondent was asked how many relatives of that type lived to be at least 10 years old and how many were ever alcoholics or problem drinkers. An alcoholic or problem drinker was defined for the respondent in a manner consistent with the DSM–IV criteria for alcohol use disorders:

By alcoholic or problem drinker, I mean a person who has physical or emotional problems because of drinking; problems with a spouse, family, or friends because of drinking; problems at work because of drinking; problems with the police because of drinking—like drunk driving—or a person who seems to spend a lot of time drinking or being hung over.

In a test-retest study conducted in conjunction with the pretest for the NLAES, the family history items generally showed good to excellent reliability, with kappas of 0.70 or higher for most types of first-degree relatives (e.g., 0.72 for fathers, 1.00 for mothers, 0.90 for brothers, and 0.73 for sisters). Slightly lower kappa values were obtained for sons and daughters (0.65 for each). For the purpose of this study, a respondent was classified as FHP if any first-degree relative of the respondent was reported as having been alcoholic or a problem drinker.

## Summary of Findings

Of the entire NLAES sample, 66 percent (*n* = 27,616) were classified as lifetime drinkers, including current (*n* = 18,352) and former (*n* = 9,264) drinkers. Approximately 50.6 percent (*n* = 13,990) of the lifetime drinkers were male and 49.4 percent (*n* = 13,626) were female. With respect to the racial distribution, 11.1 percent (*n* = 3,062) of lifetime drinkers were black and 88.9 percent (*n* = 24,554) were white. Among the lifetime drinkers in the NLAES study, classification according to both race and gender resulted in 12,518 (45.3 percent) white males; 12,036 (43.6 percent) white females; 1,472 (5.3 percent) black males; and 1,590 (5.8 percent) black females.

Tables 1 through 3 present the lifetime prevalence of alcohol dependence by age of drinking onset (for ages ≤13 and ≥21), race, and gender. For the total sample of lifetime drinkers, prevalence of lifetime alcohol dependence decreased substantially with increasing age at drinking onset, regardless of family history of alcoholism (see table 1). However, the prevalence of lifetime dependence generally was far greater among FHP respondents than among FHN respondents. For example, the prevalence of lifetime dependence among respondents who began drinking at age 21 or older was two to three times greater among those classified as FHP compared with those classified as FHN. All the trends noted for the total sample of drinkers also were observed for each race-gender subgroup of lifetime drinkers (see tables 2 and 3). Furthermore, consistent with the distribution of dependence among lifetime drinkers, the prevalence of lifetime alcohol dependence was greater among whites than among blacks and greater among male respondents than among female respondents at almost all ages of onset of drinking, regardless of family history of alcoholism.

## Discussion

As expected from previous studies, a family history of alcoholism in this analysis was shown to have a substantial effect on the development of alcohol dependence over the life span. In addition, the age at onset of drinking was a powerful predictor of lifetime alcohol dependence, regardless of family history status, race, or gender. Although these findings highlight the importance of early onset drinking and a family history of alcoholism in the development of subsequent alcohol dependence, they cannot explain why or how these two factors relate to alcohol dependence.

Another significant finding of this study is that early onset drinking cannot be viewed solely as a marker or early indicator of a family history of alcoholism (i.e., not only FHP respondents but also FHN began to drink early and therefore were at increased risk for lifetime alcohol dependence). For both FHP and FHN respondents, the likelihood of lifetime alcohol dependence decreased with increasing age at drinking onset. Those findings indicate that early onset drinking implies an increased risk of dependence, regardless of family history, and that people who drink at an early age are not necessarily destined to become alcohol dependent by virtue of having a positive family history. Moreover, a family history of alcoholism may be an indicator of shared or common environmental factors; genetic influences; or, more likely, a combination of both. This suggests that a family history of alcoholism may be, at least in part, a modifiable risk factor. The extent to which this is true, however, will have to be determined in future studies specifically aimed at clarifying and defining the contributions of environmental and genetic influences that are manifested in consistent findings of familial aggregation of alcoholism.

## Figures and Tables

**Table 1:** Age at First Alcohol Use and the Prevalence of Lifetime Alcohol Dependence by Race and Family History of Alcoholism

Prevalence of Lifetime Dependence						
Age at First Alcohol Use (years)	White		Black		Total	

	FHP^1^	FHN^1^	FHP	FHN	FHP	FHN
≤ 13	57.9 (2.2)	26.6 (2.3)	51.5 (7.7)	23.4 (7.0)	57.3 (2.1)	26.4 (2.1)
14	51.9 (2.9)	31.1 (2.7)	27.0 (8.4)	34.9 (9.0)	50.4 (2.8)	31.4 (2.6)
15	47.3 (2.3)	33.1 (2.0)	35.0 (7.3)	22.0 (5.4)	46.5 (2.2)	32.3 (1.9)
16	37.3 (1.6)	26.3 (1.2)	32.1 (6.1)	20.4 (3.8)	36.9 (1.5)	25.9 (1.2)
17	33.7 (1.7)	18.8 (1.2)	35.2 (6.0)	14.9 (3.1)	33.9 (1.6)	18.5 (1.1)
18	23.4 (1.1)	13.3 (0.7)	17.9 (3.1)	11.7 (1.9)	22.9 (1.0)	13.1 (0.6)
19	22.1 (1.8)	13.5 (1.3)	17.4 (5.0)	12.7 (4.0)	21.6 (1.7)	13.4 (1.2)
20	15.6 (1.7)	9.2 (0.9)	19.1 (4.6)	9.6 (3.1)	15.9 (1.6)	9.2 (0.9)
≥ 21	15.3 (0.9)	6.4 (0.5)	18.1 (2.6)	6.9 (1.2)	15.6 (0.8)	6.5 (0.4)

NOTE: Standard errors appear in parentheses; prevalences are presented as weighted figures.

1 FHP = family history positive; FHN = family history negative.

**Table 2:** Age at First Alcohol Use and the Prevalence of Lifetime Alcohol Dependence by Race and Family History of Alcoholism Among Males

Prevalence of Lifetime Dependence						
Age at First Alcohol Use (years)	White Male		Black Male		Total Male	

	FHP^1^	FHN^1^	FHP	FHN	FHP	FHN
≤ 13	58.8 (2.8)	30.4 (2.9)	58.8 (10.9)	21.7 (8.8)	58.8 (2.8)	29.7 (2.7)
14	56.9 (3.9)	33.3 (3.4)	20.8 (8.6)	30.9 (10.6)	54.7 (3.8)	33.2 (3.2)
15	50.6 (3.2)	33.6 (2.4)	42.4 (9.8)	21.7 (6.1)	50.1 (3.1)	32.7 (2.3)
16	40.8 (2.1)	27.8 (1.5)	45.3 (8.7)	25.1 (5.2)	41.1 (2.1)	27.7 (1.4)
17	40.8 (2.3)	19.6 (1.5)	43.6 (8.8)	15.5 (4.1)	41.1 (2.3)	19.4 (1.4)
18	28.1 (1.7)	15.9 (0.9)	17.1 (4.1)	12.9 (2.6)	27.1 (1.6)	15.7 (0.9)
19	27.8 (2.8)	15.9 (1.9)	20.7 (6.6)	16.9 (5.7)	27.1 (2.6)	16.1 (1.8)
20	22.6 (2.9)	10.9 (1.4)	20.8 (8.2)	17.4 (5.7)	22.5 (2.8)	11.6 (1.4)
≥ 21	20.1 (1.6)	8.3 (0.8)	21.2 (4.1)	8.4 (1.2)	20.3 (1.5)	8.4 (0.7)

NOTE: Standard errors appear in parentheses; prevalences are presented as weighted figures.

1 FHP = family history positive; FHN = family history negative.

**Table 3:** Age at First Alcohol Use and the Prevalence of Lifetime Alcohol Dependence by Race and Family History of Alcoholism Among Females

Prevalence of Lifetime Dependence						
Age at First Alcohol Use (years)	White Female		Black Female		Total Female	

	FHP^1^	FHN^1^	FHP	FHN	FHP	FHN
≤ 13	56.4 (3.6)	16.4 (3.0)	39.4 (10.6)	27.5 (10.2)	54.9 (3.4)	17.4 (2.9)
14	43.9 (4.0)	26.2 (4.1)	37.0 (10.9)	51.2 (14.9)	43.5 (3.9)	27.3 (4.0)
15	42.5 (3.2)	32.1 (3.4)	22.6 (9.4)	22.9 (9.6)	41.4 (3.1)	31.7 (3.3)
16	32.3 (2.2)	23.1 (1.8)	18.2 (5.1)	7.0 (3.5)	31.2 (2.1)	22.3 (1.7)
17	25.1 (2.0)	16.9 (1.8)	23.9 (6.9)	13.4 (5.3)	25.1 (1.9)	16.7 (1.7)
18	17.8 (1.3)	8.7 (0.8)	19.6 (4.8)	9.3 (2.5)	17.9 (1.3)	8.7 (0.8)
19	16.1 (1.9)	10.2 (1.4)	13.1 (7.7)	4.5 (2.4)	15.8 (1.9)	9.7 (1.3)
20	8.7 (1.5)	6.7 (1.1)	18.0 (5.3)	1.3 (0.8)	9.8 (1.5)	6.0 (0.9)
≥ 21	12.4 (0.9)	4.7 (0.5)	16.2 (3.2)	5.1 (1.3)	12.8 (0.9)	4.7 (0.4)

NOTE: Standard errors appear in parentheses; prevalences are presented as weighted figures.

1 FHP = family history positive; FHN = family history negative.

## Footnotes

1 For a definition of this and other technical terms used in this article, see glossary, p. 148.

2 This means that the 52 largest PSUs were automatically included in the study.

## GLOSSARY

Cluster sampling: A sampling method in which each sampling unit is a collection of persons, units, or elements of interest.
Kappa: A coefficient that serves as a measure of *test-retest reliability*. A kappa of 1.0 indicates that in all cases both the test and the retest produce the same result.
Oversampling: A sampling technique used to bolster the numbers of low-prevalence subgroups of the population in order to achieve adequate numbers suitable for statistical analysis.
Primary sampling units: Comprehensive, mutually exclusive categories, consisting of all persons, units, or elements of interest, usually identified in the first stage of a multistage sampling design. For example, *primary sampling units* can consist of geographic regions of the United States (e.g., cities) defined in terms of sociodemo-graphic criteria.
Selected with certainty: This typically refers to the selection of sampling units with a probability of 1.0. For example, if *primary sampling units* are designated to be selected in proportion to their size, it follows that the largest of the units will be selected with certainty.
Selected with probability: This typically refers to the selection of sampling units according to predetermined probabilities. For example, *primary sampling units* may be selected that have probabilities proportional to size (i.e., larger primary sampling units have a greater probability of being selected than do smaller primary sampling units).
Simple random sample: A method of drawing samples such that each person, element, or unit has an equal probability of being selected.
Stratification: The classification of all persons, units, or elements of interest into comprehensive, mutually exclusive categories.
Test-retest reliability: A measure of the likelihood with which two independent tests of the same variable will produce the same result.
Variance estimation procedures: A technique that allows estimation of the amount of variability (i.e., dispersion) around a measure of data, such as a percentage or mean.
Weighted percentage: Percentages that have been adjusted to account for all aspects of the sample design (e.g., different rates of selection, or *oversampling*).
